# Successful Late Emergency Cerclage for Painless Cervical Dilation in Triplet Gestation

**DOI:** 10.1155/crog/4323993

**Published:** 2026-08-21

**Authors:** Zagdon Keidar Shir, Spiegel Efrat, Silberstein Tali

**Affiliations:** ^1^ Department of Obstetrics and Gynecology, Soroka University Medical Center, Faculty of Health Sciences, Ben-Gurion University of the Negev, Beer-Sheva, Israel, bgu.ac.il

**Keywords:** asymptomatic cervical dilation, emergency cerclage, triplet pregnancy

## Abstract

**Introduction:**

The efficacy of emergency cerclage placement in triplet pregnancies remains controversial. This case describes a favorable outcome following such an intervention in prolonging gestation and improving neonatal outcomes in high‐risk multiple gestations complicated by cervical insufficiency.

**Case Presentation:**

We report a bichorionic triamniotic (BCTA) triplet pregnancy in a primigravida admitted at 21 + 5 weeks gestation with asymptomatic cervical dilation and bulging membranes. After thorough counseling regarding risks and benefits, an emergency McDonald cerclage was placed at 22 + 4 weeks. The pregnancy was prolonged until 29 + 3 weeks, when the patient underwent cesarean delivery of three healthy neonates following preterm premature rupture of membranes and suspected chorioamnionitis. Informed consent was obtained from the patient for publication.

**Discussion:**

Despite the inherent risks, emergency cerclage may be considered in highly selected triplet pregnancies with cervical insufficiency. This case underscores the importance of individualized decision‐making and may contribute to the existing literature and generate hypotheses for future research.

## 1. Introduction

Triplet pregnancies are associated with a significantly increased risk for maternal and neonatal complications, with preterm birth being the most common cause of neonatal morbidity and mortality [1, 2]. Various interventions have been investigated to reduce the risk of preterm birth in triplets, but none have been proven efficacious [3, 4]. Previous studies showed that prophylactic cerclage placement in triplet gestation did not demonstrate significant prolongation of pregnancy or improvement in neonatal outcome [5–7].

Emergent cerclage is defined as that performed in the setting of asymptomatic cervical dilation with bulging or “hourglass” membranes. Reported complications include rupture of membranes, chorioamnionitis, cervical lacerations, and suture displacement [8]. Evidence regarding the effectiveness of emergent cervical cerclage in prolonging triplet pregnancies with cervical dilation or bulging membranes is scarce.

## 2. Case Presentation

### 2.1. Informed Consent

Informed consent was obtained from the patient for publication of this case report and accompanying figure.

### 2.2. Patient Information

A 28‐year‐old primigravida with a bichorionic triamniotic (BCTA) pregnancy, conceived by in vitro fertilization, presented at 21 + 5 weeks gestation with asymptomatic cervical dilation. She was treated with vaginal progesterone tablets upon admission. Prenatal screening tests were normal. The patient had no relevant medical, familial, or psychosocial history and no prior uterine surgery. There were no known genetic risks. The IVF conception followed unexplained infertility. Following the transfer of two embryos during IVF treatment, one embryo underwent monozygotic splitting, while the second embryo developed as a singleton, resulting in a BCTA triplet pregnancy.

### 2.3. Clinical Findings

**Figure 1:**
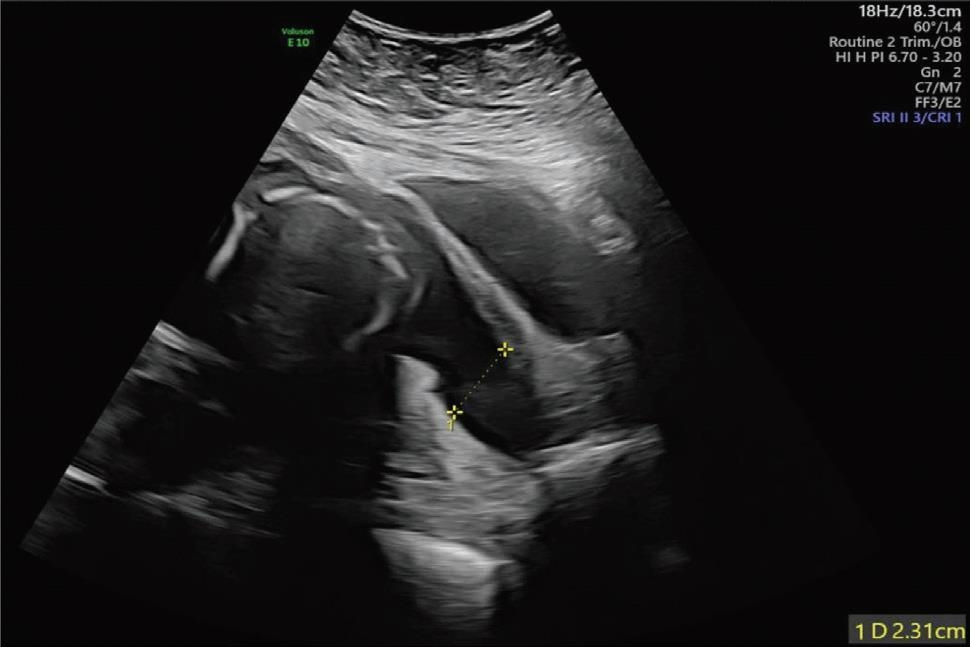
Precerclage ultrasound at 22 + 2 weeks demonstrating cervical dilation with bulging membranes reaching the level of the external cervical os.

The patient was admitted to the Maternal‐Fetal Medicine Unit at Soroka University Medical Center in January 2024. The estimated fetal weights upon admission were 528, 448, and 537 g, with a 15% discordance between the monochorionic twins. Cervical dilation of approximately 2 cm with bulging membranes was noted on physical examination. Transvaginal ultrasound performed at 22 + 2 weeks demonstrated cervical dilation of approximately 2 cm, with bulging membranes extending to the level of the external cervical os (Figure 1). Urine culture was positive for *Escherichia coli*, and the vaginal swab was positive for Group B *Streptococcus*. She was treated with an antibiotic regimen of cefuroxime for 7 days.

### 2.4. Diagnostic Assessment

The diagnosis of cervical insufficiency was based on physical examination and ultrasound findings. No imaging beyond obstetric ultrasound was required. Differential diagnoses such as preterm labor or uterine anomaly were excluded clinically.

### 2.5. Therapeutic Intervention

The patient was counselled regarding the possible management options, either conservative management or an interventional approach. She was informed about the limited data on the success rates of interventional approaches and the risks associated with cervical cerclage placement at this gestational age, including membrane rupture and infection. However, given her poor prognosis at that time and her strong desire for intervention, a decision was made to proceed with cervical cerclage placement.

**Figure 2:**
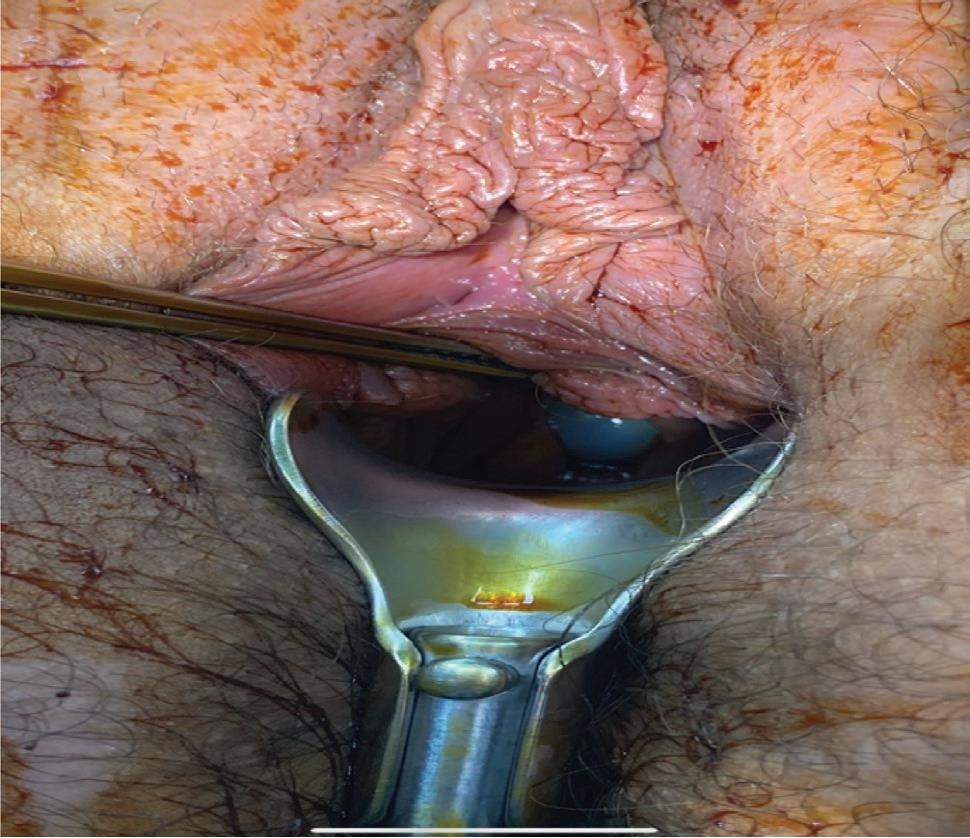
Intraoperative image before cerclage placement demonstrating bulging membranes at the external cervical os.

At 22 + 4 weeks of gestation, a McDonald cervical cerclage was inserted. Intraoperative findings included an enlarged uterus consistent with 23–24 weeks of gestation, a cervix dilated to approximately 2 cm, with bulging membranes reaching the level of the external cervical os. The estimated residual cervical tissue by physical examination was minimal (Figure 2).

The patient was placed in a steep Trendelenburg position. Gentle traction was applied to the cervix, and with the aid of gravity, the membranes retracted slightly, allowing safe placement of the cerclage. A McDonald cerclage was placed using a purse‐string technique with suture placed at the 12, 2, 4, 7 and 10 o′clock positions. The suture was tied at the 12 o′clock position.

**Figure 3:**
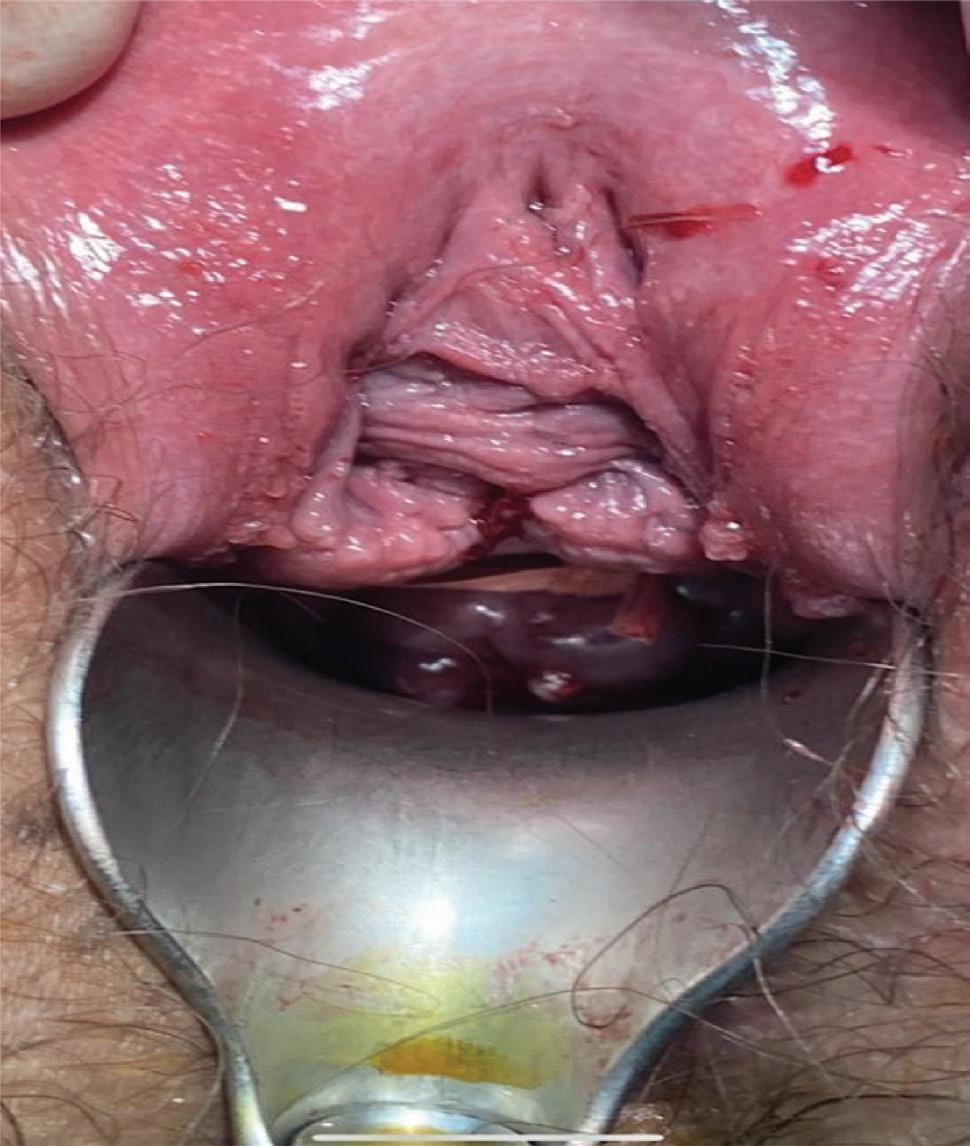
Intraoperative image following McDonald cerclage placement.

An intraoperative image following cerclage placement is presented in Figure 3. At the end of the procedure, no bulging membranes were apparent. Ultrasound assessment confirmed the presence of three viable fetuses with normal amniotic fluid volumes, and no immediate complications were observed.

### 2.6. Follow‐up and Outcomes

**Figure 4:**
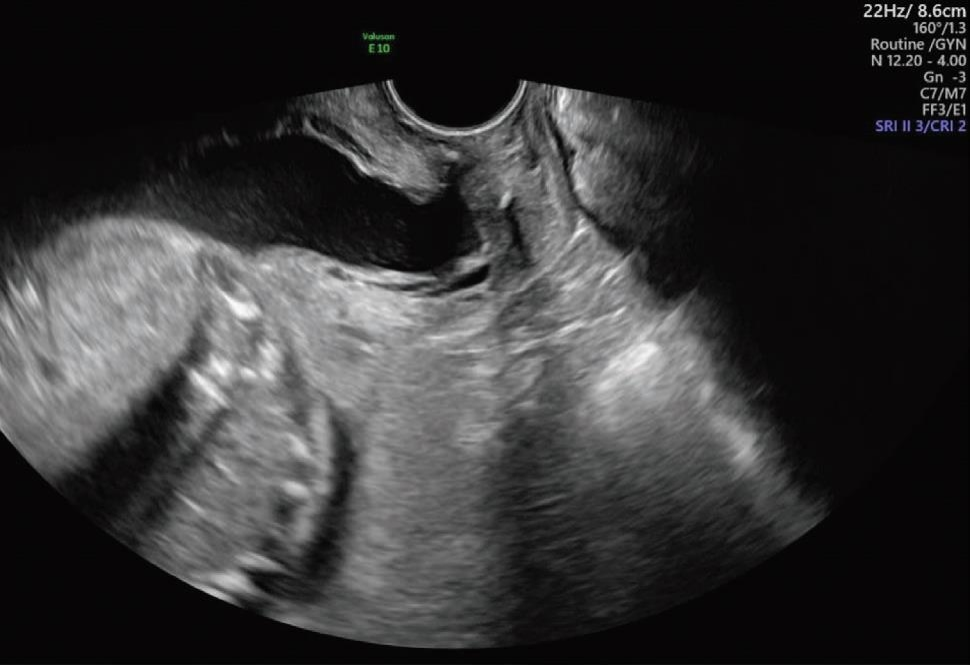
Follow‐up transvaginal ultrasound at 23 + 3 weeks demonstrating the cerclage in place, with membranes positioned above the level of the suture.

A follow‐up transvaginal ultrasound performed at 23 + 3 weeks demonstrated the cerclage in place, with the membranes positioned above the level of the suture (Figure 4).

She remained hospitalized until 26 + 4 weeks of gestation for close monitoring, infection surveillance, and continuation of supportive care. Infection surveillance included serial clinical assessments and repeated laboratory inflammatory markers, as well as vaginal swabs and urine cultures performed both prior to cerclage placement and repeatedly during hospitalization until her readmission at 28 + 6 weeks of gestation.

During the pregnancy, the patient received two courses of antenatal corticosteroids at 24 and 28 weeks of gestation.

At 28 + 6 weeks of gestation, she was readmitted to the hospital due to suspected preterm premature rupture of membranes and uterine contractions. She was transferred to the labor and delivery unit, where she was treated with antibiotics and magnesium sulfate for neuroprotection.

The cerclage was removed due to increased cervical pressure, concern for cervical tear, and suspected chorioamnionitis.

Following removal of the cerclage, cervical dilation progressed, and she ultimately delivered by cesarean section at 29 + 3 weeks of gestation.

All newborns were admitted to the neonatal intensive care unit and required minimal respiratory intervention.

The timeline is as follows:

• 21 + 5 weeks: Admission with 2 cm cervical dilation and bulging membranes.
• 22 + 4 weeks: Emergency cerclage placement.
• 24 and 28 weeks: Antenatal corticosteroids administered.
• 28 + 6 weeks: Readmission with suspected PPROM and contractions. Also treated with antibiotics and magnesium sulfate for neuroprotection.
• 29 + 3 weeks: Cesarean delivery of three viable neonates.

## 3. Discussion

This case report describes the successful use of emergency cerclage in a BCTA triplet pregnancy complicated by asymptomatic cervical dilation at 21 + 5 weeks of gestation with positive vaginal and urine cultures. Delivery of three healthy neonates at 29 + 3 weeks highlights a possible role for emergency cerclage in carefully selected pregnancies at high risk for extremely preterm delivery due to cervical insufficiency.

Prophylactic cerclage, particularly without a history of cervical insufficiency or in cases of asymptomatic cervical shortening, has not demonstrated efficacy in improving outcomes in triplet pregnancies [3, 6, 7]. Expectant management is generally the preferred approach, reflecting the limited preventive options available for these patients [4].

Emergency cerclage is generally indicated in cases of sonographic shortening of the cervix or cervical dilation upon physical examination, where the prognosis without intervention is exceedingly poor.

Evidence from twin pregnancies has shown that emergency cerclage reduced spontaneous preterm birth before 28 weeks by 40% and extended the latency period by approximately 5 weeks [9]. A meta‐analysis of 3239 participants evaluating emergency cerclage in singleton and twin pregnancies highlighted its effectiveness in improving survival rates and prolonging gestation, with fewer deliveries before 28 weeks compared to expectant management [10]. Moreover, studies indicated that cerclage placement based on physical examination findings in twin pregnancies significantly decreased the risk of preterm birth and reduces neonatal morbidity [11–15].

Although studies on cervical cerclage in triplet pregnancies are limited, existing literature regarding twin pregnancies under similar clinical conditions provided a rationale for considering its potential benefits in triplet gestations.

Research on triplet pregnancies presented conflicting evidence regarding the efficacy of cerclage placement. While some studies suggested cerclage may prolong gestational age at delivery [16–18], others reported no significant reduction in preterm birth rates [3, 8, 17, 6]. Similarly, the impact on neonatal outcomes remained inconclusive; certain studies reported no improvement [3, 6, 7, 19, 20], while others highlighted benefits in reducing preterm birth and improving neonatal health [18, 19].

Notably, studies concluding that cerclage is ineffective in triplet pregnancies often involved conditions and populations differing from our case. For instance, the largest study by Gustavo et al., which analyzed 7038 triplet pregnancies and concluded that cerclage may not prevent preterm birth or improve neonatal outcomes, focused on women with specific risk factors, such as a history of preterm birth or history of abortions. In contrast, our case involved emergent cerclage placement based solely on physical examination findings. Additionally, while the study by Gustavo et al. reported increased neonatal respiratory interventions in the cerclage group, it is unclear whether antenatal corticosteroid therapy was administered—a critical point, given its well‐documented benefits in improving neonatal respiratory outcomes [21, 22].

This case suggests a possible role of emergency cerclage in managing triplet pregnancies complicated by asymptomatic cervical dilation. Although delivery at 29 weeks remains preterm, it carries substantially lower risks compared to extremely preterm births. Without cerclage, it is possible that the pregnancy may have resulted in earlier delivery, with a heightened risk of severe neonatal morbidity and mortality [10, 23, 24].

This case illustrates a favorable outcome following emergency cerclage in a highly selected clinical scenario. However, this single case does not allow for generalization and should be interpreted with caution. The observed outcome may reflect patient selection, timing of intervention, and absence of overt infection at presentation. This report may contribute to the existing literature and generate hypotheses for future research.

## 4. Strengths and Limitations

The strength of this case lies in the detailed clinical course and the favorable outcome. Limitations include its single‐patient nature, which inherently limits generalizability, and the lack of long‐term developmental follow‐up. Moreover, the absence of a comparative group further restricts interpretation of the finding.

## 5. Patient Perspective

The patient expressed that despite her initial fears, she was grateful to have been fully informed and involved in the decision‐making. She felt emotionally supported and shared that she was “thankful every day” for the positive outcome of her triplets.

## Author Contributions

Dr. Shir Zagdon‐Keidar had full access to all data and takes responsibility for the integrity of the data and the accuracy of the data analysis.

## Funding

This research received no specific grant. The authors declare that no funding sources had any role in study design, data collection, analysis, interpretation, or manuscript preparation.

## Disclosure

All authors have read and approved the final version of the manuscript. Dr.Shir Zagdon‐Keidar affirms that this manuscript is an honest, accurate, and transparent account of the case being reported, that no important aspects have been omitted, and that any discrepancies have been explained.

## Consent

Informed consent was obtained from the patient for publication of this case report and accompanying figures.

## Conflicts of Interest

The authors declare no conflicts of interest.

## Data Availability

All data supporting the findings of this study are included within the article.

## References

[1] Chibber R. , Fouda M. , Shishtawy W. , Al-Dossary M. , Al-Hijji J. , Amen A. , and Mohammed A. T. , Maternal and Neonatal Outcome in Triplet, Quadruplet and Quintuplet Gestations Following ART: a 11-Year Study, Archives Of Gynecology and Obstetrics. (2013) 288, no. 4, 759–767, 10.1007/s00404-013-2796-x, 23543239. 23543239

[2] Hruby E. , Sassi L. , Görbe É. , Hupuczi P. , and Papp Z. , The Maternal and Fetal Outcome of 122 Triplet Pregnancies, Orvosi Hetilap. (2007) 148, no. 49, 2315–2328, 10.1556/oh.2007.28119, 18048111. 18048111

[3] Young C. M. , Stanisic T. , Wynn L. B. , Shrivastava V. L. , Haydon M. L. , and Wing D. A. , Use of Cerclage in Triplet Pregnancies With an Asymptomatic Short Cervix, Journal Of Ultrasound In Medicine. (2014) 33, no. 2, 343–347, 10.7863/ultra.33.2.343, 24449739. 24449739

[4] Moragianni V. A. , Aronis K. N. , and Craparo F. J. , Biweekly Ultrasound Assessment of Cervical Shortening in Triplet Pregnancies and the Effect of Cerclage Placement, Ultrasound In Obstetrics & Gynecology. (2011) 37, no. 5, 617–618, 10.1002/uog.8905, 21154786. 21154786

[5] Rafael T. J. , Berghella V. , Alfirevic Z. , and Cochrane Pregnancy and Childbirth Group , Cervical Stitch (Cerclage) for Preventing Preterm Birth in Multiple Pregnancy, Cochrane Database of Systematic Reviews. (2014) 2014, no. 9, 10.1002/14651858.CD009166.pub2. PMC10629495 25208049

[6] Bernasko J. , Lee R. , Pagano M. , and Kohn N. , Is Routine Prophylactic Cervical Cerclage Associated With Significant Prolongation of Triplet Gestation?, Journal of Maternal-Fetal & Neonatal Medicine. (2006) 19, no. 9, 575–578, 10.1080/14767050600825607, 16966127. 16966127

[7] Rebarber A. , Roman A. S. , Istwan N. , Rhea D. , and Stanziano G. , Prophylactic Cerclage in the Management of Triplet Pregnancies, American Journal Of Obstetrics And Gynecology. (2005) 193, no. 3, 1193–1196, 10.1016/j.ajog.2005.05.076, 16157136. 16157136

[8] ACOG Practice Bulletin No.142: Cerclage for the Management of Cervical Insufficiency, Obstetrics and Gynecology. (2014) 123, no. 2, 372–379, 10.1097/01.AOG.0000443276.68274.cc, 24451674. 24451674

[9] Zeng C. , Liu X. , Zhao Y. , Pei C. , Fu Y. , Wang W. , Li Y. , He L. , and Zhang W. , Pregnancy Outcomes and Factors Affecting the Clinical Effects of Emergency Cerclage in Twin Pregnancies With Cervical Dilation and Prolapsed Membranes, International Journal of Gynecology & Obstetrics. (2022) 157, no. 2, 313–321, 10.1002/ijgo.13774, 34076897. 34076897

[10] Hulshoff C. C. , Bosgraaf R. P. , Spaanderman M. E. A. , Inthout J. , Scholten R. R. , and Van Drongelen J. , The Efficacy of Emergency Cervical Cerclage in Singleton and Twin Pregnancies: A Systematic Review With Meta-Analysis, American journal of obstetrics & gynecology MFM. (2023) 5, no. 7, 100971, 10.1016/j.ajogmf.2023.100971, 37084870. 37084870

[11] Tan H. , The Use of Cervical Cerclage in Asymptomatic Twin Pregnancies With Cervical Shortening or Dilation: A Twelve-Year Retrospective Cohort Study, BMC Pregnancy Childbirth. (2023) 23, no. 1, 10.1186/s12884-023-06013-6, 37773110. PMC10540472 37773110

[12] Ponce J. , Benítez L. , Baños N. , Goncé A. , Bennasar M. , Muñoz M. , Cobo T. , and Palacio M. , Latency to Delivery in Physical Examination-Indicated Cerclage in Twins Is Similar to That in Singleton Pregnancies, International Journal of Gynecology & Obstetrics. (2022) 159, no. 1, 188–194, 10.1002/ijgo.14070, 34890050. 34890050

[13] Roman A. , Zork N. , Haeri S. , Schoen C. N. , Saccone G. , Colihan S. , Zelig C. , Gimovsky A. C. , Seligman N. S. , Zullo F. , and Berghella V. , Physical Examination-Indicated Cerclage in Twin Pregnancy: A Randomized Controlled Trial, American Journal of Obstetrics And Gynecology. (2020) 223, no. 6, 902.e1–902.e11, 10.1016/j.ajog.2020.06.047, 32592693. 32592693

[14] D’Antonio F. , Eltaweel N. , Prasad S. , Flacco M. E. , Manzoli L. , and Khalil A. , Cervical Cerclage for Prevention of Preterm Birth and Adverse Perinatal Outcome in Twin Pregnancies With Short Cervical Length or Cervical Dilatation: A Systematic Review and Meta-Analysis, PLoS Medicine. (2023) 20, no. 8, 10.1371/journal.pmed.1004266. PMC10456178 37535682

[15] Abbasi N. , Barrett J. , and Melamed N. , Outcomes Following Rescue Cerclage in Twin Pregnancies, Journal of Maternal-Fetal & Neonatal Medicine. (2018) 31, no. 16, 2195–2201, 10.1080/14767058.2017.1338260, 28597706. 28597706

[16] Sumners J. E. , Moore E. S. , Ramsey C. J. , and Eggleston M. K. , Transabdominal Cervical Cerclage in Triplet Pregnancies and Risk of Extreme Prematurity and Neonatal Loss, Journal of Obstetrics and Gynaecology. (2011) 31, no. 2, 111–117, 10.3109/01443615.2010.542512, 21281022. 21281022

[17] Vilchez G. , Kumar K. , Singh S. , Patel N. , Kao M. , Lagos M. , and Duncan J. , Risk of Preterm Birth and Neonatal Outcomes After Cerclage Placement in Triplet Pregnancies, Journal of Maternal-Fetal & Neonatal Medicine. (2022) 35, no. 25, 5409–5415, 10.1080/14767058.2021.1881476, 33847210. 33847210

[18] Goldman G. A. , Dicker D. , Peleg D. , and Goldman J. A. , Is Elective Cerclage Justified in the Management of Triplet and Quadruplet Pregnancy?, Australian and New Zealand Journal of Obstetrics and Gynaecology. (1989) 29, no. 1, 9–11, 10.1111/j.1479-828X.1989.tb02867.x, 2562615. 2562615

[19] Elimian A. , Figueroa R. , Nigam S. , Verma U. , Tejani N. , and Kirshenbaum N. , Perinatal Outcome of Triplet Gestation: Does Prophylactic Cerclage Make a Difference?, Journal of Maternal‐Fetal Medicine. (1999) 8, no. 3, 119–122, https://pubmed.ncbi.nlm.nih.gov/10338066/. 10338066 10.1002/(SICI)1520-6661(199905/06)8:3<119::AID-MFM9>3.0.CO;2-O

[20] Moragianni V. A. , Cohen J. D. , Smith S. J. , Rosenn M. F. , and Craparo F. J. , The Role of Ultrasound-Indicated Cerclage in Triplets, Ultrasound in Obstetrics and Gynecology. (2009) 34, no. 1, 43–46, 10.1002/uog.6387, 19565536. 19565536

[21] Jobe A. H. , Goldenberg R. L. , and Kemp M. W. , Antenatal Corticosteroids: An Updated Assessment of Anticipated Benefits and Potential Risks, American Journal of Obstetrics And Gynecology. (2024) 230, no. 3, 330–339, 10.1016/j.ajog.2023.09.013, 37734637. 37734637

[22] McGoldrick E. , Stewart F. , Parker R. , and Dalziel S. R. , Antenatal Corticosteroids for Accelerating Fetal Lung Maturation for Women at Risk of Preterm Birth, Cochrane Database of Systematic Reviews. (2021) 12, no. 2, 10.1002/14651858.CD004454.pub4. PMC8094626 33368142

[23] Ehsanipoor R. M. , Seligman N. S. , Saccone G. , Szymanski L. M. , Wissinger C. , Werner E. F. , and Berghella V. , Physical Examination-Indicated Cerclage, Obstetrics and gynecology. (2015) 126, no. 1, 125–135, 10.1097/AOG.0000000000000850. 26241265

[24] Alfirevic Z. , Stampalija T. , and Medley N. , Cervical Stitch (Cerclage) for Preventing Preterm Birth in Singleton Pregnancy, Cochrane Database of Systematic Reviews. (2017) 4, no. 6. 10.1002/14651858.CD008991.pub3 PMC6481522 28586127

